# Self-Crossing Leads to Weak Co-Variation of the Bacterial and Fungal Communities in the Rice Rhizosphere

**DOI:** 10.3390/microorganisms9010175

**Published:** 2021-01-15

**Authors:** Jingjing Chang, Shaohua Shi, Lei Tian, Marcio F. A. Leite, Chunling Chang, Li Ji, Lina Ma, Chunjie Tian, Eiko E. Kuramae

**Affiliations:** 1 Key Laboratory of Mollisols Agroecology, Northeast Institute of Geography and Agroecology, Chinese Academy of Sciences, Changchun 130102, China; changjingjing@iga.ac.cn (J.C.); sshua2013@163.com (S.S.); tianlei@iga.ac.cn (L.T.); changchunling@iga.ac.cn (C.C.); jili@iga.ac.cn (L.J.); malina23@163.com (L.M.); 2 University of the Chinese Academy of Sciences, Beijing 100049, China; 3 Department of Microbial Ecology, Netherlands Institute of Ecology NIOO-KNAW, 6708 PB Wageningen, The Netherlands; M.Leite@nioo.knaw.nl; 4 Ecology and Biodiversity, Institute of Environmental Biology, Utrecht University, 3584 CH Utrecht, The Netherlands

**Keywords:** microbial ecology, *Oryza rufipogon*, *Oryza sativa*, co-occurrence

## Abstract

The rhizomicrobial community is influenced by plant genotype. However, the potential differences in the co-assembly of bacterial and fungal communities between parental lines and different generations of rice progenies have not been examined. Here we compared the bacterial and fungal communities in the rhizomicrobiomes of female parent *Oryza rufipogon* wild rice; male parent *Oryza sativa* cultivated rice; their F1 progeny; and the F2, F3 and F4 self-crossing generations. Our results showed that the bacterial and fungal α-diversities of the hybrid F1 and self-crossing generations (F2, F3, F4) were closer to one of the two parental lines, which may indicate a role of the parental line in the diversity of the rhizosphere microbial community assembly. Self-crossing from F1 to F4 led to weak co-variation of the bacterial and fungal communities and distinct rhizosphere microbiomes. In the parental and self-crossing progenies, the reduction of community dissimilarity was higher for the fungal community than for the bacterial community.

## 1. Introduction

Rice is the main source of food for half the world’s population and remains a very important economic crop [1]. The area of rice cultivation has been decreasing due to the scarcity of available arable land and the competition for other land uses. The area of rice cultivation in the world in 2019 was 162,055,938 hectares according to the Food and Agriculture Organization of the United Nations database, which was reduced by 3,466,546 hectares compared to that in 2018 [2]. The reduction of rice area cultivation combined with the population growth has resulted in the development of rice varieties with high yields and strong stress resistance to enhance rice productivity. The development of cultivated rice from wild rice via breeding programs has significantly altered the genetic repertoire of the rice genome to increase germination rates, yield and nutrition levels [3], but wild rice genes associated with survival, such as those related to disease resistance and lodging resistance, have been lost [4,5]. In addition to the genetic features underlying the tolerance of rice to biotic and abiotic stresses, the microbial community inhabiting the rhizosphere may contribute to plant health and growth by providing key functions in protection against pathogen infection, nutrient acquisition and abiotic tolerance [6,7,8,9]. In rice, nitrogen-use efficiency has been linked to the recruitment of distinct root microbiota, and the nitrogen-use efficiency of the “indica” variety is superior to that of the “japonica” variety [10].

The rhizosphere is a complex micro-ecosystem formed by plant growth and the molecules absorbed and secreted by plants and differs from that of bulk soil [11,12]. In the rhizosphere, soil, plants and microorganisms interact with each other [13]. Rhizosphere microbes affect crop productivity through interactions with crop signal transduction pathways [14,15,16,17,18]. Tian et al. [19] identified differences in the community structure of root-associated bacteria between wild rice and cultivated rice using a denaturing gradient gel electrophoresis (DGGE)-based technique, and Shenton et al. [20] reported that the diversity of bacterial communities associated with different *Oryza* wild and cultivated rice generations was only weakly correlated with phylogenetic distance. Wild rice, a perennial, was reported to have longer root length, higher plant height and better resistance to numerous biotic and abiotic stresses than cultivated rice, such as lodging and drought tolerance [21,22,23]. In comparison, cultivated rice, an annual, was shown to have high root biomass, more lateral roots and higher yield than wild rice [24,25]. Additionally, both wild rice (*Oryza ruffipogen*) and cultivated rice (*Oryza sativa*) are diploid species with 24 chromosomes with AA genome lineage, easily hybridized, exhibit normal meiotic chromosome pairing and can generate viable fertile hybrids [26]. Recent evidence demonstrates that the structures of bacterial communities in the rhizospheres of wild and cultivated rice are significantly different [27]. However, little attention has been given to the structure of microbial communities in different generations of rice progenies rhizosphere. In addition to plant-bacteria interactions, bacterial–fungal interactions in the rhizosphere are part of the communication network that maintains microhabitat balance. Consequently, assessments of the dynamics of the co-assembly of bacterial and fungal communities are of great relevance [28]. However, such studies are scarce, and no study has examined the co-assembly of bacterial and fungal communities in different generations of rice progenies. Thus, the main objective of this study was to explore the composition, structure, co-assembly and co-occurrence of the bacterial and fungal communities in two rice parental lines; their F1 progeny; and the F2, F3 and F4 self-crossing generations. We hypothesized that the bacterial and fungal communities in the rhizosphere co-vary and that their homogeneity increases with successive rice self-crossing. To test this hypothesis, we used *Oryza rufipogon* wild rice as the female parent and a local breed of *Oryza sativa* cultivated rice as the male parent to obtain the hybrid F1 generation and subsequent F2, F3 and F4 generations by self-crossing. The rhizosphere bacterial and fungal communities of the parental lines and their progeny were analyzed by amplicon sequencing of the partial 16S rRNA gene and the internal transcribed spacer (ITS), respectively.

## 2. Materials and Methods

### 2.1. Plant Material and Rhizosphere Samples

Dongxiang wild rice (Chinese common wild rice, *Oryza rufipogon*) is the most precious wild rice germplasm resource in northern China. Its drought, cold and pest resistance make it superior to any other wild rice line in China [29]. We used *Oryza rufipogon* wild rice as the female parent and a local breed of *Oryza sativa* cultivated rice as the male parent to obtain the hybrid F1. Then, the F1 individuals were self-crossed to obtain F2, which was self-crossed to obtain F3. Finally, F3 was self-crossed to obtain F4. The parental lines were provided by the Jiangxi Academy of Agricultural Sciences. Experimental plots for the rice F1, F2, F3 and F4 generations were set up in an open greenhouse of the Jiangxi Academy of Agricultural Sciences (28°56′N, 115°95’E) in Nanchang, Jiangxi Province, China. The open greenhouse protected the plots from unfavorable weather conditions, such as large temperature differences between morning and evening and heavy rainfall. All the seeds of each species include parental lines and generations (F1, F2, F3 and F4) were well-mixed and 120 seeds were randomly selected. The hulls were removed from the seeds, and 120 seeds of each species were disinfected by immersion in 2% sodium hypochlorite solution for 10 min. The seeds were then washed three times for 1 min each in sterile water. The disinfected seeds were placed on filter paper (90 cm diameter) in plate dishes and incubated for 48 h at 25 °C. The seedlings were transplanted to the plots in the open greenhouse, each plot was 9 (3 × 3) square meters. Different parental lines and generations seedlings were transfer to different plot according to the line spacing of 20–30 cm, 3–5 seedlings per hill. Three to five seedlings which may include different genotypes were randomly transferred to one hill. Nitrogen (70 kg P ha^−1^ as urea), phosphorus (50 kg P ha^−1^ as single superphosphate), potassium (50 kg K ha^−1^ as muriate of potash) and zinc (5 kg Zn ha^−1^ as zinc sulfate heptahydrate) were applied and incorporated in all plots 1 day before transplanting. The field was flooded 2 days after transplanting, and a floodwater depth of 3–5 cm was maintained until 10 days before full heading developmental stage, when the field was drained. In order to let the rice grow naturally, no insecticides or herbicides were used. In August 2017, when the plants were 90 days old (full heading developmental stage), the rice plants and soil were collected: five replicates of rice rhizosphere soil were collected per plot of the parental lines and hybrids, and three replicates of bulk soil were collected. In each replicate, five plants were selected and mixed together as one replicate. Rhizosphere soil was collected by removing the soil closely adhered to the roots using a sterilized fine brush and stored at −80 °C [30].

### 2.2. Measurements of Soil Physical and Chemical Properties

Soil pH and electrical conductivity (EC) were measured in a 1:2.5 mixture of soil:water as described by Luo et al. [31]. The soil organic carbon (SOC) content was determined by the potassium dichromate volumetric method [32] and then converted to soil organic matter (SOM) using a factor of 1.724 following the methods proposed by Pribyl et al. [33]. Soil total nitrogen (TN) was measured by the Kjeldahl method, and total phosphorus (TP) was measured according to a soil analysis manual [34]. Available phosphorus (AP) and available potassium (AK) were determined via the NaHCO_3_ extraction method and ammonium acetate extraction method, respectively [35]. The soil pH, EC, SOM, TN, TP, AP and AK were 5.85, 96.53 μS cm^−1^, 38.99 g kg^−1^, 0.83 g kg^−1^, 378.09 mg kg^−1^, 17.62 mg kg^−1^ and 26.41 mg kg^−1^, respectively.

### 2.3. DNA Extraction and Amplicon Sequencing

A 0.5 g sample of rhizosphere soil was ground into powder in liquid nitrogen, and DNA was extracted from the sample according to the instructions of the Fast DNA SPIN Kit (MP Biomedicals, Eschwege, Germany). DNA concentrations were measured using a NanoDrop 2000 spectrophotometer (NanoDrop Technologies, Inc.; Wilmington, DE, USA). The extracted DNA was used as the template for PCR. We amplified the V3-V4 hypervariable region of bacterial rRNA 16S gene by using the primers 338F (ACTCCTACGGGAGGCAGCA) and 806R (GGACTACHVGGGTWTCTAAT) and amplified fungal ITS1 by using the primers ITF5F (GGAAGTAAAAGTCGTAACAAGG) and ITS1R (GCTGCGTTCTTCATCGATGC). Sample-specific 7-bp barcodes were incorporated into the primers for multiplex sequencing. Each PCR contained 5 μL of Q5 reaction buffer (5×), 5 μL of Q5 High-Fidelity GC buffer (5×), 0.25 μL of Q5 High-Fidelity DNA Polymerase (5U μL^−1^), 2 μL (2.5 mM) of dNTPs, 1 μL (10 µM) each of forward and reverse primer, 2 μL of DNA template and 8.75 μL of ddH2O. Thermal cycling consisted of initial denaturation at 98 °C for 2 min, followed by 25 cycles of denaturation at 98 °C for 15 s, annealing at 55 °C for 30 s and extension at 72 °C for 30 s. A final extension was performed for 5 min at 72 °C. The PCR amplicons were purified with Agencourt AMPure Beads (Beckman Coulter, Indianapolis, IN, USA) and quantified using the PicoGreen dsDNA Assay Kit (Invitrogen, Carlsbad, CA, USA). After the individual quantification step, the amplicons were pooled in equal amounts, and paired-end 2 × 300 bp sequencing was performed using the Illumina MiSeq platform with the MiSeq Reagent Kit v3 at Shanghai Personal Biotechnology Co.; Ltd. (Shanghai, China).

### 2.4. Bioinformatics and Statistical Analysis

Amplicon sequences with a length of less than 150 bp, ambiguous bases or mononucleotide repeats of >8 bp were discarded by using USEARCH 6.1 in QIIME (Quantitative Insights Into Microbial Ecology, v1.8.0, http://qiime.org/) [36]. Paired-end reads were assembled using FLASH [37]. After chimera detection, the remaining high-quality sequences were clustered into operational taxonomic units (OTUs) at 97% sequence identity by UCLUST [38]. A representative sequence was selected from each OTU using default parameters. OTU taxonomic classification was conducted by BLAST searching the representative sequence set against the SILVA database (version 132, https://www.arb-silva.de/aligner/) and UNITE database (version 5.0, https://unite.ut.ee/analysis.php/) [39,40]. In order to ensure the reliability and accuracy of the analysis results, OTUs representing less than 0.001% of total sequences across all samples were discarded. To minimize the differences in sequencing depth across samples, an averaged, rounded rarefied OTU table was generated by averaging 100 evenly resampled OTU subsets under a minimum sequencing depth of 90% for further analysis [41]. The bacterial and fungal Illumina raw sequence data have been deposited in the National Center for Biotechnology Information Sequence Read Archive (NCBI SRA) database (http://www.ncbi.nlm.nih.gov/sra) under accession numbers SRP168367 and SRP168829.

The MicrobiomeAnalyst Platform (https://www.microbiomeanalyst.ca) was used to calculate the α-diversity and perform linear discriminant effect size (LEfSe) analysis [42]. Alpha diversity is a comprehensive indicator reflecting richness and evenness and was calculated using OTUs. The Chao1 and Shannon indices were used to reflect richness and diversity, respectively [43]. In order to ensure the reliability and accuracy of the analysis results, we removed OTUs with abundance values of less than 0.001% of the total sequenced samples [41].

One-way ANOVA with linear discriminant analysis (LDA) was performed in SPSS 17.0 to test whether there were significant differences in the microbial communities among *Oryza rufipogon* wild rice (female parent), *Oryza sativa* cultivated rice (male parent), and the hybrid F1 and self-crossing F2, F3 and F4 generations (*p* < 0.01) [44]. LEfSe analysis is an LDA-based method in which the LDA score is used to identify the main key communities that differ significantly among different groups at the family taxonomic level [45]. It emphasizes both statistical significance and biological relevance, allowing identifying differentially abundant features that are also consistent with biologically meaningful categories. Specifically, the non-parametric factorial Kruskal–Wallis sum-rank test was used to detect features with significant differential abundance; biological significance is subsequently investigated using a set of pairwise tests among subclasses using the Wilcoxon rank-sum (unpaired) test. As a last step, LEfSe uses linear discriminant Analysis to estimate the effect size (LDA score) of each differentially abundant feature. Then we obtained the key bacterial and fungal family of each samples and statistic the proportion of the phylum which the main bacterial and fungal family subordinated.

Between-class analysis (BCA) and co-inertia analysis were applied to explore the effects of the parental rice lines and rice generations on the microbial community (bacterial and fungal communities) variability and covariance using the package ade4 R v3.1.1. BCA evaluates the percentage of variability in the microbiome that is explained by the different generations. Co-inertia analysis provides the percentage of covariance between the bacterial and fungal communities and thus evaluates how the changes in one community interfere with the other. Prior to the analysis, the microbiome data were transformed using Hellinger transformation [46]. To search for significant effects, both BCA and co-inertia analysis were performed using Monte Carlo permutation tests. In the Monte Caro permutation test, all values are shuffled to produce random arrangements. The software then compares how far the observations are from random, which is analogous to a *p*-value test against the null hypothesis that the rice generation has no effect on the variability of the microbiome. We used 999 random permutations [47]. The results of co-inertia analysis are presented as plots with arrows, in which the length of the arrow indicates the extent of covariance between the bacteria and fungi in each treatment: the longer the arrow, the lower the covariance between these two microbial groups. In addition, arrows projected in the same direction indicate a strong association between treatments with respect to microbial composition [48]. The bacterial and fungal community structure covariance scores are given by the co-inertia coefficient.

To determine the co-occurrence between the bacteria and fungi, we calculated SparCC’s rank correlation coefficients (Python 2.6.1) of taxonomic families. This method randomly creates 100 simulation datasets from the original data and calculates the Pseudo-*p* value by determining how many of the 100 datasets produce the same order of magnitude correlation with the real data [49]. For each rice generation, 5 replicates were used to calculate the correlation coefficients. The network was visualized by Gephi 8.0 [Spearman’s r (absolute value) > 0.8, *p* < 0.01], while the psych and igraph R packages were used to calculate the network topology characteristics.

To explore the effects of self-crossing in the microbial community, we evaluated how the community dissimilarity changed along generations via generalized dissimilarity modeling (GDM). GDM is a statistical approach for analyzing community composition turnover. The community composition is given by a dissimilarity index, in our case the Bray–Curtis dissimilarity. The GDM method has an advantage that can account for the mean-variance relationship in the dissimilarity metric and avoid the bias imposed by the Bray–Curtis dissimilarity [50]. GDM was originally designed to model spatial variation in biodiversity between pairs of geographical locations but can also accommodate special types of biological variation, such as relationships between generations. First, we assumed that after four generations of self-crossing, the genetic variability was reduced from F1 to F4. Based on that assumption, we established levels of genetic variability ranging from lowest in F4 (0) to highest in F1 (3). These levels were used in GDM to evaluate whether the differences between generations can explain the dissimilarity in the microbiome. Community turnover is measured by the partial ecological distance, which corresponds to the increase in the dissimilarity index following an increase in levels of genetic variability (from generation F4 to F1). GDM uses l-splines to more accurately estimate the increase in the partial ecological distance and evaluate the increase in community dissimilarity. We opted not to include the parental lines (*Oryza rufipogon* and *Oryza sativa*) in the analysis of self-crossing, given that their genetic variability is not the same. We performed GDM analysis for the total microbial community (bacteria and fungi) and separately for the bacterial and fungal communities using the package “gdm” [51].

## 3. Results

### 3.1. Structures of Bacterial and Fungal Communities in Different Generations of Rice Progenies Rhizosphere

**Figure 1:**
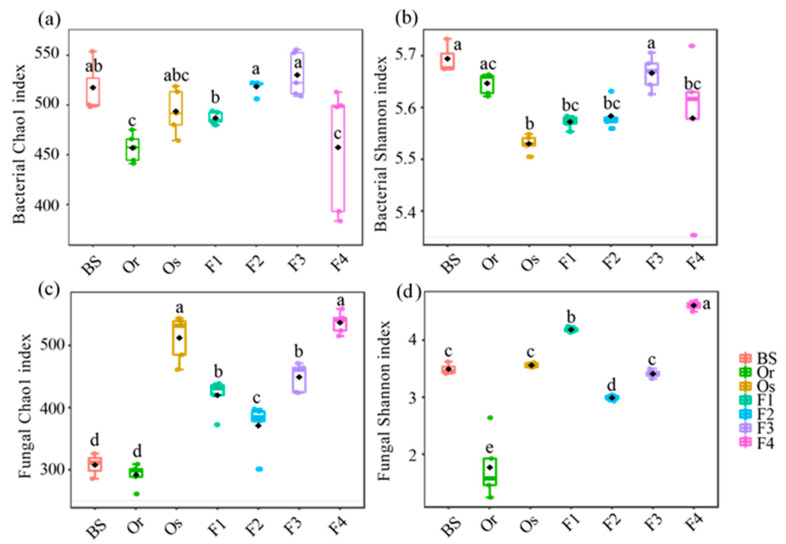
The α-diversity of the (**a**,**b**) bacterial and (**c**,**d**) fungal communities in bulk soil (BS) and the rhizospheres of the female parent *Oryza rufipogon* (Or) wild rice; male parent *Oryza sativa* (Os) cultivated rice; hybrid generation (F1); and the generations (F2, F3 and F4) obtained by self-crossing. Data with the same letters within each column indicate no significant difference by one-way ANOVA with LSD tests at *p* < 0.05.

To reflect the number of species in the microbial community, including their abundance and diversity, we calculated the α-diversity. The bacterial Chao1 indices of F1, F2 and F3 were closer to that of *Oryza sativa* than those of *Oryza rufipogon* (Figure 1a). The bacterial Shannon indices of the hybrid generations (F1, F2 and F4) were similar to that of *Oryza sativa*, and the Shannon index of F3 was similar to that of *Oryza rufipogon* (Figure 1b). With respect to fungal α-diversity, the Chao1 and Shannon indices of the hybrid generations (F1, F2, F3 and F4) were closer to those of *Oryza sativa*. Among the hybrid and self-crossing generations, F2 had the lowest fungal α-diversity (both the Chao1 and Shannon indices), and there was an upward trend from F3 to F4 (Figure 1c,d).

**Figure 2:**
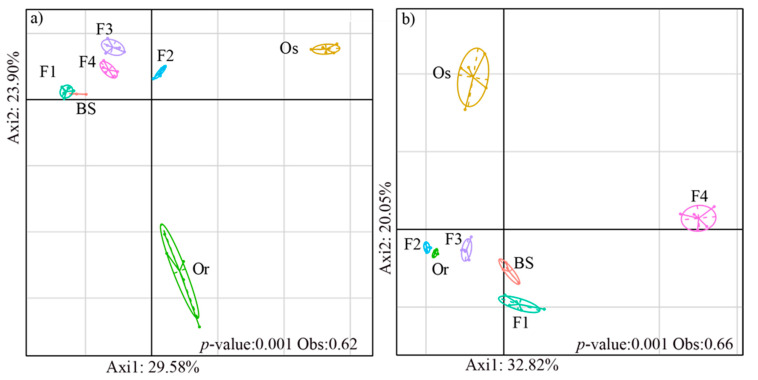
Between-class analysis (BCA) of (**a**) bacterial and (**b**) fungal communities in bulk soil (BS) and the rhizospheres of the female parent *Oryza rufipogon* (Or) wild rice; male parent *Oryza sativa* (Os) cultivated rice; hybrid generation (F1); and the generations F2, F3 and F4 obtained by self-crossing. Each rice generation is represented by a circle, the size of each circle represents the 95% confidence interval.

To explore the diversity of species composition in the microbial communities of the rice rhizosphere among different generations, we performed BCA analysis. The results indicate that the bacterial and fungal community structures in the rhizospheres were significantly different (*p* < 0.01) among the rice generations (including the parental lines and hybrid generations) (Figure 2). The bacterial communities of bulk soil, F1, F2, F3 and F4 grouped together, and those of the parental lines *Oryza rufipogon* and *Oryza sativa* each formed separate clusters (Figure 2a) (*p* < 0.01). The percentages of variation explained by axis1 and axis2 were 29.58% and 23.90%, respectively. The fungal communities of *Oryza rufipogon*, F2 and F3 formed one cluster; F1 and BS formed another cluster; and F4 and *Oryza sativa* clustered individually (Figure 2b). The results of BCA analysis also showed that the differences among generations explained a higher proportion of fungal variability (66%) than bacterial variability (62%).

We used LEfSe analysis to identify the microorganisms underlying these structural differences in bacterial and fungal communities among the parental lines and the hybrid and self-crossing rice generations. The results indicated that there were 70 bacterial families significant different in rhizosphere among the parental and rice generations based on the LDA score and corrected *p*-value (false discovery rate, FDR) [46] (Table S1). The cumulative proportion of bacteria in the total microbial population selected by LEfSe analysis decreased in the order *Oryza rufipogon* (62.95%) > *Oryza sativa* (60.47%) > F2 (59.41%) > F1 (52.45%) > F3 (52.11%) > F4 (51.40%). We found that the abundances of *Nitrospiraceae*, *Hydrogenophilaceae* and *Xanthobacteraceae* in the rhizospheres of the hybrid and self-crossing generations were closer to those in the rhizosphere of *Oryza rufipogon* than those in the rhizosphere of *Oryza sativa* (Figures S1–S4; Tables S1 and S2). *Frankiaceae* in the rhizospheres of the hybrid and self-crossing generations were closer to those in the rhizosphere of *Oryza rufipogon*, while there was no *Frankiaceae* in the rhizosphere of *Oryza sativa*. *Anaerolineaceae*, *Archangiaceae*, *Nocardioidaceae* and *Mycobacteriaceae* were less abundant in the rhizospheres of the hybrid and self-crossing generations, which were more similar to those of *Oryza sativa*. Most of the bacteria in the rhizospheres of parental lines and these generations belong to the phyla *Proteobacteria* and *Actinobacteria* (Figure S5a). For the rhizosphere fungi, 87 significant differences (at the family level) were observed among the rice generations. The cumulative proportion of fungi in the total microbial population selected by LEfSe analysis decreased in the order *Oryza sativa* (62.98%) > F1 (42.66%) > F4 (39.40%) > F3 (19.58%) > *Oryza rufipogon* (14.07%) > F2 (8.71%). The abundances of *Lasiosphaeriaceae*, *Cucurbitariaceae*, *Didymosphaeriaceae* and *Diversisporaceae* in the rhizospheres of the hybrid and self-crossing generations were closer to those in the rhizosphere of *Oryza rufipogon*, and the abundances of *Mycosphaerellaceae*, *Ustilaginaceae* and *Hydnodontaceae* in the rhizospheres of the hybrid and self-crossing generations were closer to those in the rhizosphere of *Oryza sativa* (Figures S3 and S4). Most of the fungi in the rhizospheres of parental lines and these generations belong to the phyla *Ascomycota* and *Basidiomycota* (Figure S5b). These results indicated that the phylum which the key bacterial and fungal family subordinated remains unchanged in parental lines and their generations.

### 3.2. Co-Assembly of Bacterial and Fungal Communities in Different Generations of Rice Progenies Rhizosphere

**Figure 3:**
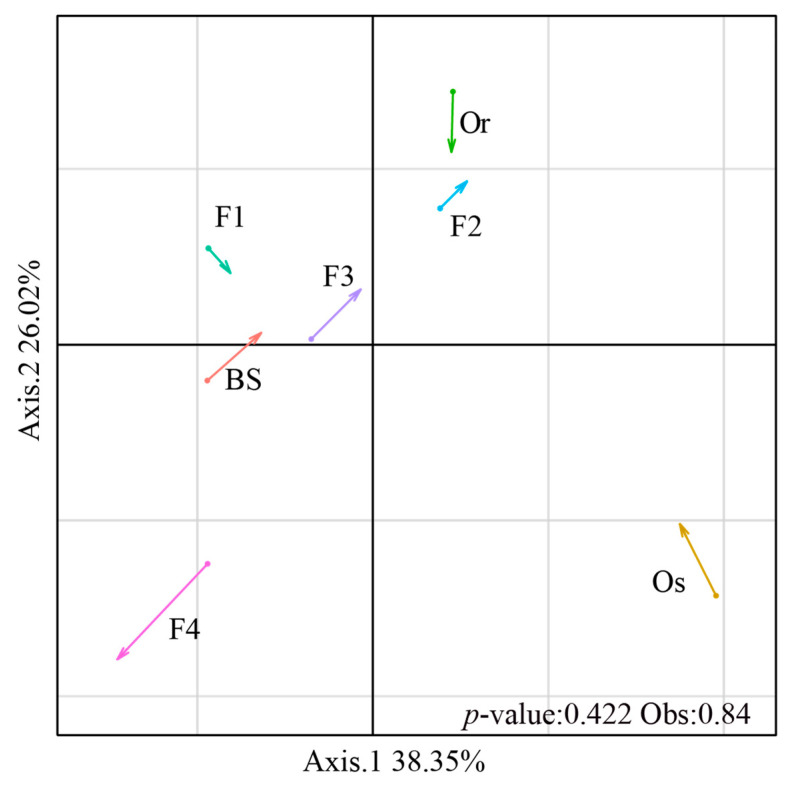
Co-inertia analysis of bacterial and fungal communities in bulk soil (BS) and the rhizospheres of the female parent *Oryza rufipogon* (Or) wild rice; male parent *Oryza sativa* (Os) cultivated rice; hybrid generation (F1); and the generations F2, F3 and F4 obtained by self-crossing. The arrows represent the co-variation of both communities within the generations (the shorter the arrow, the higher the covariance between bacterial and fungal community). The arrows origins represent the bacterial communities and the arrow heads represent the positions of the fungal communities in the co-inertia space.

To determine the dissimilarities in bacterial and fungal community structures between the parental lines and rice generations, we performed co-inertia analysis. Co-inertia analysis showed that the covariance of the bacterial and fungal communities differed among *Oryza rufipogon*, *Oryza sativa*, F1 and F4; and F2 and F3 showed similar covariance of the bacterial and fungal communities (*p* > 0.01). The length of each arrow in Figure 3 represents the covariation of both communities within the treatments (the longer the arrow, the smaller the covariance between bacterial and fungal communities in each treatment). With self-crossing from F1 to F4, the arrow size increased (Figure 3), which indicates that the covariance between the bacterial and fungal communities decreased.

**Figure 4:**
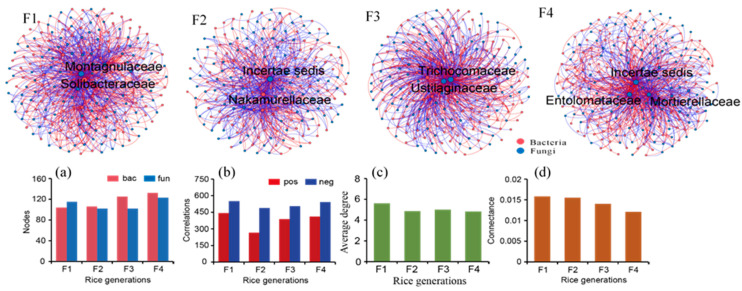
Network based on SparCC correlation coefficients (SparCC’s rho cut-off = 0.8, *p* < 0.01) showing the co-occurrence patterns of parental-affected groups of bacteria and fungi in different rice generations. Red and blue lines represent significant positive and negative (*p* < 0.01) linear relationships, respectively. The rhizospheres of the hybrid generation (**F1**) and the progenies obtained by self-crossing (**F2**, **F3**, **F4**) were analyzed. Number of nodes (**a**), number of correlations (**b**), average degree (**c**) and connectance (**d**) of network for rhizosphere microbial communities for the rice progenies F1–F4.

To determine the co-occurrence of families of bacteria and fungi in the rhizospheres of the hybrid (F1) and self-crossing (F2, F3, F4) generations, SparCC analysis (SparCC’s rho cut-off = 0.8, *p* < 0.01) was performed. The co-occurrences of bacteria and fungi were most complex in F2, F3 and F4 (Figure 4d). In the hybrid F1 and self-crossing generations F2, F3, and F4, the number of nodes (bacteria and fungi plus together) followed the order F4 > F3 > F1 > F2. There were more negative correlations than positive correlations in the rhizosphere of F1, which had the largest connectance values among the rice generations (F1, F2, F3 and F4) (Figure 4). The number of positive correlations decreased in the order F1 (443) > F4 (412) > F3 (389) > F2 (265); similarly, the number of negative correlations followed the order F1 (551) > F4 (542) > F3 (505) > F2 (488). The average degree of the network was highest in F1, followed by F3, F2 and F4. The pink nodes in Figure 4 represent bacteria in the network that had the largest connection degrees in F1. The blue nodes represent fungi in the network that had the largest connection degrees in F2, F3 and F4. The number of connections was largest for *Montagnulaceae* and *Caulobacteraceae* in F1, *Nakamurellaceae* in F2, *Trichocomaceae* and *Ustilaginaceae* in F3, *Entolomataceae* and *Mortierellaceae* in F4.

**Figure 5:**
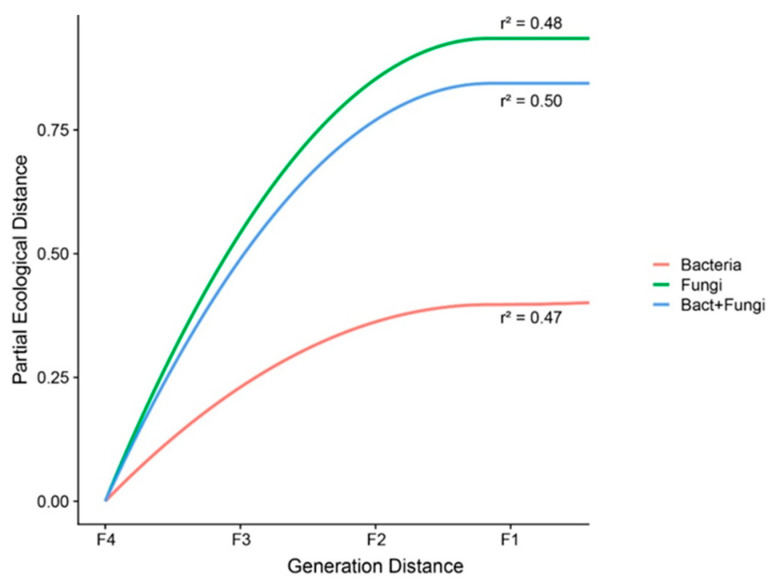
Increasing community dissimilarity of the bacterial (red), fungal (green) and total (bacteria and fungi) (blue) communities according to the generations of rice self-crossing as given by the generalized dissimilarity model. The maximum height reached by each curve reveals the total amount of compositional turnover associated with the generations of self-crossing. The shape of each function indicates the rate of compositional turnover.

To explore the effects of self-crossing in the bacterial and fungal communities, we evaluated the changes in the dissimilarity of the communities (bacteria and fungi) along rice generations via GDM. The results revealed that rice self-crossing reduced the microbial community dissimilarity, but the magnitude of the decrease differed for bacteria and fungi. The fungal community dissimilarity showed a higher sensitivity to self-crossing than the bacterial community and total community (bacteria + fungi) (maximum partial ecological distances of 0.94, 0.44 and 0.84 for the fungal, bacterial and total communities, respectively; see Figure 5). In summary, the greater the number of self-crossings, the smaller the dissimilarity within the bacterial and fungal communities in the rice rhizosphere.

## 4. Discussion

Dongxiang wild rice *Oryza rufipogon*, which was used in this study as the female parent line, has genetic features conferring resistance to drought, cold and pests that make it superior to any other wild rice line in China [30]. This species is also the ancestor of the local breed of cultivated rice *Oryza sativa* that we used as the male parent line. The bacterial richness indices (Chao1 index) of F1, F2 and F3 were closer to those of *Oryza sativa*, as were the fungal richness and diversity of the hybrid generations (F1, F2, F3 and F4). Our results showed that the diversities of bacteria and fungi were largely influenced by *Oryza sativa* parent cultivated rice.

The bacterial community structures of the bulk soil and rhizospheres of F1, F2, F3 and F4 grouped together, whereas those of *Oryza rufipogon* and *Oryza sativa* formed separate clusters. For the fungal communities, *Oryza rufipogon*, F2 and F3 formed a cluster, and F1 and bulk soil formed another cluster, whereas the rhizosphere fungal communities of F4 and *Oryza sativa* clustered individually. Plant genetic selectivity is one of the main factors for guiding the rhizosphere microbial communities, which drives changes in the communities and relative abundances of bacteria and fungi [52]. The rhizosphere microbial populations of the hybrid and self-crossing generations may be driven by either the female or male parent lines. We found that the bacterial families *Nitrospiraceae* and *Hydrogenophilaceae* were influenced by the parent *Oryza rufipogon* wild rice and were associated with the hybrid and self-crossing generations, which have been implicated in carbon-nitrogen–phosphorus cycling [53,54,55,56]. An interesting finding was that *Frankiaceae* in the rhizospheres of the hybrid and self-crossing generations were closer to those in the rhizosphere of *Oryza rufipogon*, while there was no *Frankiaceae* in the rhizosphere of *Oryza sativa*. The *Frankiaceae* represent a group of microbes with nitrogen-fixation potential [57]. On the other hand, the fungal families *Lasiosphaeriaceae* and *Cucurbitariaceae* were associated with the hybrid and self-crossing generations. Previous studies reported that microbes from these family groups are able degrade cellulose and promote root development [58,59,60]. Future studies may explore the growth promotion potential of these groups in rice plants.

We also found evidence that the bacterial and fungal groups that were more abundant in the rhizosphere of the *Oryza rufipogon* wild rice female parent, i.e., *Nitrospiraceae* and *Hydrogenophilaceae*, may have beneficial effects on rice growth, and some fungi (*Mycosphaerellaceae* and *Ustilaginaceae*) more abundant in the rhizosphere of the *Oryza sativa* cultivated rice male parent could be potential plant pathogens [61,62,63]. For the male cultivated parent *Oryza sativa*, most of the more abundant bacteria in the rhizosphere represented by *Anaerolineaceae* and *Nocardioidaceae*, have been described to degrade carbohydrates and insoluble compounds [56,64,65,66,67]. An interesting finding in our study was that *Anaerolineaceae* showed an upward trend among F1, F2, F3 and F4, which suggests that *Anaerolineaceae* increased with self-crossing and was more abundant in the rhizosphere of *Oryza sativa* cultivated rice, the male parent. *Anaerolineaceae*, a representative group of *Chloroflexi*, is an important microorganism involved in methane metabolism. Nonetheless, with successive self-crossing, the rice traits became more homogeneous, with lower genetic diversity and greater enrichment of specific target microorganisms such as *Anaerolineaceae* in the rhizosphere microbiome. Most of the key bacteria identified by LEfSe analysis in the current study belong to the phyla *Proteobacteria* and *Actinobacteria*, corroborating with previous studies that the rhizosphere microbiome is dominated by bacterial families or genera belonging to *Proteobacteria* and *Actinobacteria* in cultivated crop accessions, including in common bean [68,69,70], barley [5], lettuce [71] and sunflower [72]. The above findings suggested that domestication maintained key bacterial families belonging to *Proteobacteria* and *Actinobacteria* phyla.

As demonstrated by co-inertia analysis, with successive self-crossing from F1 to F4, the co-variation of the bacterial and fungal communities decreased (increase the size of the arrow in Figure 3). Shenton et al. [20] found that the phylogeny of root-associated microbiome weakly correlated with *Oryza* phylogeny; however, the authors used Bray–Curtis dissimilarity rhizosphere microbiome, a method that has bias towards abundance analysis [50]. Besides, the differences in plant age, soil nutrient status and primers could explain the incongruent results between Shenton et al. [20] and the current study. According to Edwards et al. [73], microbiome composition of rice varied with genotype and soil source under controlled conditions (greenhouse). Since soil source was controlled in the current study, we managed to identify differences between the plant genotypes and between parental lines and self-crossing. Cao et al. [74] found that the content of chlorophyll, the relative steady phase of chlorophyll, the net photosynthetic rate, the active photosynthetic duration and the leaf source capacity per leaf area in the cultivated rice were higher than those in wild rice, suggested that the grain yield of wild rice was lower than that of cultivated rice. Mohammadkhani et al. [75] investigated that the contents of soluble sugar and proline can significantly improve plants response to stress. The soluble sugar contents and the proline contents in wild rice were higher than those in cultivate rice, which suggested that the stress tolerance of wild rice was stronger than that of cultivated rice [19]. Studies have suggested that the F2 generations hold stronger cold tolerance than other generations, possibly due to the higher contents of soluble sugar and proline [76,77]. The recombination of rice genetic material by self-crossing may affect the levels of root-exuded metabolites [78], which ultimately affects the rhizomicrobiome composition. This hypothesis can be tested in future study.

We explored microbial interactions via two different approaches. First, we considered the co-occurrences of the whole microbiome in each rice generation (F1–F4) as a proxy of potential taxon-taxon interaction. We observed a decrease in the number of positive co-occurrences in generation F2 (Figure 4b), suggesting a loss of commensalism and mutualism [79,80]. We also observed a reduction of connectance along generations F1–F4 (Figure 4d), suggesting weak co-dependence between different microbes. Second, we performed co-inertia analysis to investigate the covariance between the bacterial and fungal communities. In contrast to the previous co-occurrence analysis, which focused on the overall interaction of the two communities, this analysis focused on their co-dependence. The co-inertia analysis confirmed that self-crossing reduced the covariance between the bacterial and fungal communities in the rice rhizosphere. Taken together, these results indicated a weakening of microbial community co-dependence with self-crossing, which was mainly attributable to a loss of interactions between bacteria and fungi.

Moreover, when we investigated the specific effects of self-crossing on microbial community dissimilarity, we observed that the reduction of community dissimilarity was higher for the fungal community than for the bacterial community. BCA analysis confirmed this result by revealing that a higher proportion of fungal community variability could be explained by the differences in the generations of self-crossing. The increasing dissimilarity along generations together with the reduced co-inertia and overall connectance suggest that self-crossing impacted rhizosphere microbial community selection and disrupted the co-dependence within the microbiome [47,81].

Self-crossing is a widely used technique to study changes in rice characteristics. However, studies on the impact of self-crossing in the rhizosphere microbiome remain scarce. Our study contributed to this topic by showing that self-crossing reduced the dissimilarity of both bacterial and fungal communities together with a weakened co-dependence between those two groups of microbes. Surprisingly, generation F2, expected to present the highest genetic variability, did not present a highly distinct rhizosphere microbiome [82,83]. A better understanding on the mechanisms behind this loss of dissimilarity will require a better understanding on the phylogenetic distance between the plant generations together with a description of the plant’s exudates. This is left as an avenue for future studies.

In agreement with our findings, Pérez-Jaramillo et al. [70] reported a reduction in the complexity of microbial co-occurrence when comparing domestic and wild beans. Our study extends this finding to rice by showing that generations of self-crossing selected a more distinct microbiome that was less co-dependent. Consequently, compared with the parent lines, generations of self-crossing led to a less complex and more independent microbial community in the rice rhizosphere. F2 is expected to be the most heterogenous and heterozygous and thus, more abundant and diverse rhizomicrobiome might be recruited. Surprisingly, we found that F2 genotype harbored the less complex connectance of rhizomicrobial community while is the most heterogenous and heterozygous compared to other generations. Further studies are necessary to examine the reason why F2 generation harbored the less complex connectance of rhizomicrobial community. Moreover, Schlemper et al. [12] found that different sorghum cultivars assembled significantly different bacterial community compositions. We extend their results by showing that the reduction in rice genetic diversity due to successive self-crossing increase microbial dissimilarity and weakened the co-dependence within the microbiome. Our study confirms the strong role of plant genetic variability in selecting a specific microbiome, as self-crossing led to the selection of a more fungal-specific than bacterial-specific rice rhizosphere.

## 5. Conclusions

We found that the α-diversity of the rhizospheres of the hybrid and self-crossing generations was shifted compared with the parental lines. In addition, the community structure of the bacteria and fungi in the rhizosphere differed significantly among the rice generations, and these differences were mainly related to the bacterial families *Anaerolineaceae* and *Nitrospiraceae*, and the fungal families *Lasiosphaeriaceae*, *Mycosphaerellaceae* and *Ustilaginaceae*. The rhizosphere bacterial groups affected by the wild rice parent might be involved in carbon and nitrogen metabolism, and the fungal groups affected by the wild rice parent might contribute to rice growth. The bacterial groups affected by the cultivated rice parent are mainly involved in methane metabolism (*Anaerolineaceae*), but some fungal groups (*Mycosphaerellaceae*, *Ustilaginaceae*, *Kickxellaceae* and *Helotiaceae*) could cause disease. Co-inertia analysis, BCA and GDM demonstrated that the strength of this covariance is dependent on rice generation, with stronger covariance between the bacterial and fungal communities in the hybrid F1. Self-crossing from F1 to F4 decreased the co-assembly of bacterial and fungal communities and the co-occurrence of microbes in the rice rhizosphere.

## Data Availability

The raw sequence datasets have been deposited in the NCBI SRA database (https://www.ncbi.nlm.nih.gov/) under accession numbers SRP168367 and SRP168829 for bacteria and fungi, respectively.

## Supplementary Materials

The following are available online at https://www.mdpi.com/2076-2607/9/1/175/s1. Figure S1: Rhizosphere bacterial communities (family level) affected by wild rice *Oryza rufipogon* (Or) female parent. Rhizosphere of parental wild rice *Oryza rufipogon* (Or), parental cultivated rice *Oryza sativa* (Os) and different hybrid generations of rice progenies (F1) obtained by self-crossing (F2, F3, F4), Figure S2: Rhizosphere bacterial communities (family level) affected by cultivated rice *Orysa sativa* (Os) male parent. Rhizosphere of parental wild rice *Oryza rufipogon* (Or), parental cultivated rice *Oryza sativa* (Os) and different hybrid generations of rice progenies (F1) obtained by self-crossing (F2, F3, F4). Figure S3: Rhizosphere fungal communities (family level) affected by parental wild rice *Oryza rufipogon* (Or). Rhizosphere of parental wild rice *Oryza rufipogon* (Or), parental cultivated rice *Oryza sativa* (Os) and different hybrid generations of rice progenies (F1) obtained by self-crossing (F2, F3, F4). Figure S4: Rhizosphere fungal communities (family level) affected by parental cultivated rice *Oryza sativa* (Os). Rhizosphere of parental wild rice *Oryza rufipogon* (Or), parental cultivated rice *Oryza sativa* (Os) and different hybrid generations of rice progenies (F1) obtained by self-crossing (F2, F3, F4). Figure S5: The proportion of the key bacteria family (a) and fungi family (b) affiliated to phylum. Rhizosphere of parental wild rice *Oryza rufipogon* (Or), parental cultivated rice *Oryza sativa* (Os) and different hybrid generations of rice progenies (F1) obtained by self-crossing (F2, F3, F4). Table S1: The key bacterial families with significant differences among different rice generations identified by LEfSe analysis, Table S2: The key fungal families with significant differences among different rice generations identified by LEfSe analysis.
