# Supplementary material for: Self-Crossing Leads to Weak Co-Variation of the Bacterial and Fungal Communities in the Rice Rhizosphere
Source: Microorganisms. 2021 Jan 15;9(1):175. doi: 10.3390/microorganisms9010175 (PMC7830547; doi:10.3390/microorganisms9010175)
Supplement: Supplementary file 1 [file microorganisms-09-00175-s001.pdf]

Self-crossing leads to weak co-variation of the bacterial and fungal communities in the rice rhizosphere

Supplementary Material

Table S1. The key bacterial families with significant differences among different rice generations identified by LEfSe analysis.

Bacterial family	Correct P-values	Or abundance	Os abundance	F1 abundance	F2 abundance	F3 abundance	F4 abundance	LDA score
Anaerolineaceae	0.0003	1067700	1131200	1461400	1741300	2199000	1801500	5.75
Nitrospiraceae	0.0003	389490	1120100	101160	506460	112650	240670	5.71
Hydrogenophilaceae	0.0003	346430	853700	60175	594960	124510	384560	5.6
Xanthobacteraceae	0.0003	375590	88823	803350	323610	284550	608720	5.55
Acidobacteriaceae Subgroup 1	0.0003	388420	70546	465930	381380	85692	8990.8	5.36
Bradyrhizobiaceae	0.0007	298740	107660	296480	457550	230080	118480	5.24
Archangiaceae	0.0007	451710	169920	176960	135810	118270	134110	5.22
Nocardioidaceae	0.0003	280490	2922.3	22822	0	10408	36241	5.15
Comamonadaceae	0.0003	70292	247140	0	57143	34326	28629	5.09
Mycobacteriaceae	0.0003	288660	55654	201390	98992	94667	181810	5.07
Geobacteraceae	0.0003	111790	233750	2733.8	56331	25372	3860.5	5.06
Gemmatimonadaceae	0.0004	273450	55722	213170	139940	144750	115050	5.04
Nitrosomonadaceae	0.0003	221300	121390	77428	192730	17314	65540	5.01
Burkholderiales Incertae Sedis	0.0003	0	41066	0	0	182810	0	4.96
Planctomycetaceae	0.0020	75951	36132	62990	29993	71331	199500	4.93
Syntrophaceae	0.0006	59782	191500	39208	92952	37930	30226	4.91
Nakamurellaceae	0.0003	81448	25456	10965	141560	62642	3935.3	4.84
Cellulomonadaceae	0.0007	15611	16667	0	2557.1	138320	9345.8	4.84
Kineosporiaceae	0.0003	42651	10781	130360	72493	52505	16395	4.78
Holophagaceae	0.0003	40657	119120	0	23035	15153	1980.2	4.77
Frankiaceae	0.0006	25745	963.39	109390	7725.4	17230	19894	4.73
Micromonosporaceae	0.0005	55015	9811.1	115830	17974	36951	64740	4.72
uncultured Aminicenantes	0.0004	0	96573	0	6826.8	17168	4132.2	4.68
Gallionellaceae	0.0006	42951	98743	3643.7	2571.6	28455	16876	4.68
Roseiflexaceae	0.0003	0	3882	47340	14482	93718	73556	4.67
Geodermatophilaceae	0.0020	122040	54668	66559	45228	135760	94962	4.66
Methylocystaceae	0.0007	102990	140650	157670	87165	134260	173550	4.64
uncultured Acidobacteria	0.0004	12516	95652	33693	73349	44778	95313	4.62
Caldilineaceae	0.0004	84908	28476	1842.5	11995	9522.6	30692	4.62
Haliangiaceae	0.0007	49042	31219	47476	44465	89541	15673	4.57
uncultured Chloroflexi	0.0040	71787	71458	30092	72328	104770	61663	4.57
Intrasporangiaceae	0.0003	71817	1005.5	17282	0	37151	10820	4.56
Clostridiaceae	0.0003	77312	33274	61085	5977.4	35233	58391	4.55
uncultured Caldilineae	0.0003	4200.7	65538	0	28075	14317	19724	4.52
Coriobacteriaceae	0.0005	66770	40142	2737.2	2520.5	62459	66518	4.51
Caulobacteraceae	0.0005	64862	16540	15496	33271	2855.8	12559	4.49
Rhodobiaceae	0.0010	94217	38212	100310	44399	38653	54890	4.49
Xanthomonadaceae	0.0012	9285.3	61460	14585	17090	11280	0	4.49
Roseiarcaceae	0.0011	6243.8	8759.7	8208.1	58000	13280	0	4.46
Bacillaceae	0.0004	57483	22443	61066	5940.5	18823	23896	4.44
Gaiellaceae	0.0007	26111	17723	55611	862.81	20744	41438	4.44
uncultured proteobacterium	0.0004	54180	34136	3666.5	46227	977.52	5804.4	4.42
Desulfarculaceae	0.0005	7321.3	47752	0	22191	1889.4	7941.9	4.38
Helicobacteraceae	0.0005	29386	45936	0	8578.2	3778.8	1991.4	4.36
Hyphomicrobiaceae	0.0003	0	13719	0	40073	21886	0	4.3
uncultured Anaerolineaceae	0.0005	2028.4	14715	27381	38417	37058	10878	4.26
Acidimicrobiaceae	0.0007	37590	14640	20072	846.02	29516	28582	4.26
Sporichthyaceae	0.0010	36563	0	2741.4	2579.9	0	0	4.26
Syntrophobacteraceae	0.0007	12574	35187	0	11920	912.83	11788	4.25
Streptomycetaceae	0.0011	38543	3905.5	16387	4284.2	14388	23752	4.24
Acetobacteraceae	0.0005	5185.1	34251	10930	33354	13214	0	4.23
Sphingomonadaceae	0.0003	20997	32284	0	0	0	0	4.21

Desulfobacteraceae	0.0006	3086	31363	0	20473	8478.8	7954.3	4.2
uncultured Gemmatimonadetes	0.0003	8378.9	6866.3	0	1731.2	0	30987	4.19
Rhodocyclaceae	0.0006	29168	24367	0	0	1881.8	6931.2	4.16
Xanthomonadales Incertae Sedis	0.0018	12504	21486	13676	16248	2871	25699	4.06
Fraxinus excelsior (European ash)	0.0003	0	0	0	0	21876	8839.4	4.04
Thermomonosporaceae	0.0010	2085.4	985.71	20960	0	0	9675.5	4.02
Rhizobiales Incertae Sedis	0.0036	19831	6800.7	12757	17132	10230	0	4
uncultured Bellilinea sp.	0.0132	3093.4	959.69	20071	3456.9	2864.4	2998.7	3.98
Syntrophorhabdaceae	0.0004	2142.5	11730	913.24	17057	0	0	3.93
Desulfovibrionaceae	0.0005	15680	8814.4	0	846.02	0	0	3.89
Beijerinckiaceae	0.0010	8314.6	0	5466.7	0	917.85	14791	3.87
Trebouxiophyceae sp.	0.0007	14646	3908	0	0	13464	1980.2	3.86
uncultured Parcubacteria group	0.0081	5322.7	2011.1	2754.5	12795	0	0	3.81
Draconibacteriaceae	0.0034	0	8756	0	5906.2	0	2902.2	3.64
uncultured Streptomyces sp.	0.0027	0	0	8204.8	828.5	5612.9	1980.2	3.61
Candidatus Falkowbacteria	0.0069	0	5831.1	0	6898.4	2866	0	3.54
Alcaligenaceae	0.0341	1065	0	2738.5	0	6646.6	6037.7	3.52
Total abundance		6295143	6046846	5245158	5940885	5210559	5140345	
Selected bacteria/total bacteria		62.95%	60.47%	52.45%	59.41%	52.11%	51.40%	

Table S2. The key fungal families with significant differences among different rice generations identified by LEfSe analysis.

Fungal family	Correct *P*-value	Or abundance	Os abundance	F1 abundance	F2 abundance	F3 abundance	F4 abundance	LDA score
Lasiosphaeriaceae	0.0005	161730	3967400	725870	38932	82131	233930	6.29
Pyronemataceae	0.0005	281190	382390	151800	46742	190520	1013100	5.68
Cordycipitaceae	0.0006	315860	2947.8	647830	1395.2	3175	21917	5.51
Mortierellaceae	0.0008	777.65	625.49	527490	103.37	972.97	82161	5.42
Boletaceae	0.0005	103480	15887	517460	2378.2	7099.9	73922	5.41
Cryphonectriaceae	0.0005	1931	1213	444120	192.31	194.87	12029	5.35
Trichocomaceae	0.0008	76960	71583	118420	12966	52517	327170	5.2
Chaetomiaceae	0.0005	11250	33488	52542	5785.2	12430	280480	5.14
Pleosporaceae	0.0005	481.13	269200	292.88	3009.8	0	0	5.13
Basidiobolaceae	0.0008	97958	310210	42561	240440	283440	299430	5.13
Sporormiaceae	0.0005	51467	52326	230300	15463	126020	253950	5.08
Mycosphaerellaceae	0.0005	198.27	17745	6353.4	7286.9	226970	6934.4	5.05
Ustilaginaceae	0.0005	698.35	18672	11400	83867	216660	175160	5.03
Nectriaceae	0.0005	24624	46811	230950	17708	226060	140360	5.03
Terramycetaceae	0.0005	9806.2	202530	1643	2751.8	0	3428.2	5.01
Claroideoglomeraceae	0.0005	5012.7	190910	668.92	192.31	20764	4562.8	4.98
Orbiliaceae	0.0005	187.33	167590	1829.2	153320	1274.1	0	4.92
Teloschistaceae	0.0006	15368	916.12	133250	828.9	1275.7	8630.6	4.82
Entolomataceae	0.0015	0	204.16	189.47	0	499.44	129900	4.81
Teratosphaeriaceae	0.0005	18957	37152	3598.1	5819.6	123220	754.91	4.79
Blastocladiaceae	0.0005	948.52	12296	4432.8	807.15	57188	103160	4.71
Hydnodontaceae	0.0005	5447.6	30295	71560	12872	13695	106520	4.7
Phallaceae	0.0017	198.27	1120.1	0	97.532	84568	0	4.63
Sebacinales Group B	0.0005	6708.4	33194	50153	8095.5	24937	81200	4.57
Kickxellaceae	0.0005	3306.9	10198	23366	10005	4210	59136	4.45
Lulworthiaceae	0.0006	62315	52164	25737	67134	11444	29629	4.44
Cucurbitariaceae	0.0005	3532.6	56633	10698	15026	2942.6	9584.5	4.43
Cortinariaceae	0.0005	4689.1	23627	8607.4	1498.3	30759	46742	4.35
Archaeosporaceae	0.0008	43772	1038.4	195.26	0	0	1183.8	4.34
Helotiaceae	0.0005	0	3604.8	5180.7	14979	41906	9455.4	4.32
Microthyriaceae	0.0005	0	39999	0	0	0	2641.1	4.3
Phaeosphaeriaceae	0.0005	184.83	37916	6687.1	6897.1	8933.5	107.77	4.28
Didymosphaeriaceae	0.0006	1895.6	706.33	37885	1013.9	3028.5	20894	4.27
Diversisporaceae	0.0050	10815	2225.9	9605.7	9168.7	10518	39520	4.27
Strophariaceae	0.0005	713.24	4114	32026	838.17	3732.2	20873	4.19
Gomphidiaceae	0.0005	1502.3	9155	10826	3013.7	2349.2	32580	4.19
Sebacinaceae	0.0005	774.7	4576.6	0	391.72	2074	30574	4.18
Sclerodermataceae	0.0010	878.64	26591	1922.4	15532	10360	4815.9	4.11
Pezizaceae	0.0005	0	25380	2408.9	222.48	1083.5	14258	4.1
Magnaporthaceae	0.0023	25234	9320.3	13308	12234	12328	839.77	4.09
Gigasporaceae	0.0005	0	0	292.88	0	3357.9	24282	4.08
Ophiocordycipitaceae	0.0005	1179.2	3392.9	0	0	0	21239	4.03
Clavulinaceae	0.0022	1448.7	7828.5	11764	2521.9	5700.9	20142	3.97
Pacisporaceae	0.0008	108.29	6476.2	772.93	1222.7	602.42	15879	3.9
Pseudeurotiaceae	0.0007	14731	5788	9535.5	95.411	2473.7	13748	3.86
Thelephoraceae	0.0023	920.85	1007.7	2577.9	95.411	3108	14526	3.86
Amanitaceae	0.0005	8633	13052	97.666	0	3508.8	4066.5	3.81
Heterogastridiaceae	0.0024	664.37	4348.3	390.51	1139.7	3580	13132	3.8
Ganodermataceae	0.0005	508.51	2283.9	5072.2	578.83	1553.9	12308	3.77
Clavariaceae	0.0012	198.27	3610.5	3264.3	502.33	2151.9	12098	3.77
Cantharellaceae	0.0006	0	1327.1	7972.7	11446	965.08	0	3.76
Meruliaceae	0.0025	0	1246.7	1335.7	437.09	381.68	11332	3.75
Niaceae	0.0009	108.29	10643	1150.5	1551.3	98.624	6304.1	3.72

Clavicipitaceae	0.0005	10337	0	376.01	0	0	0	3.71
Microascaceae	0.0016	962.17	2749.2	4769.3	385.89	1878.9	10728	3.71
Botryobasidiaceae	0.0010	1094	5127.1	10038	3892.2	972.52	10904	3.7
Tubeufiaceae	0.0005	2117.5	0	0	8463.2	205.95	0	3.63
Chaetosphaeriaceae	0.0129	698.35	1229.9	773.89	1291.6	1465.3	9250.9	3.63
Montagnulaceae	0.0018	2560.3	8356.4	4391.4	703.97	591.66	913.71	3.59
Endochytriaceae	0.0025	597.18	524.72	0	589.78	0	7400.7	3.57
Tetraplosphaeriaceae	0.0005	0	1883.7	1734.6	7304.3	0	0	3.56
Inocybaceae	0.0023	198.27	7142.3	2784.6	477.05	1881.2	2353	3.54
Spizellomycetaceae	0.0016	782.12	6237.2	0	1193.1	5946.3	5178	3.49
Leotiaceae	0.0008	0	202.85	6026.8	0	0	3938.1	3.48
Rhizophydiaceae	0.0006	0	5827.6	2212.8	0	886.79	1745.4	3.46
Verrucariaceae	0.0009	0	5763.9	97.666	0	492.21	0	3.46
Hymenochaetaceae	0.0010	0	2194.5	4794.7	111.24	1273.2	3650.2	3.38
Oedogoniomycetaceae	0.0007	0	513.46	95.138	0	0	4485.9	3.35
Alphamycetaceae	0.0005	0	4074.2	0	515.08	982.58	0	3.31
Boliniaceae	0.0018	484.73	0	290.06	0	0	3636.7	3.26
Trichomonascaceae	0.0005	0	0	2878.4	0	3215.3	0	3.21
Ambisporaceae	0.0025	0	933.64	0	0	0	3245	3.21
Hydnaceae	0.0016	0	297.81	3169.5	405.52	294.68	2296.7	3.2
Dacrymycetaceae	0.0033	93.664	733.63	1923.6	0	98.624	3125.3	3.19
Coniochaetaceae	0.0438	1054.5	302.31	1138.9	111.24	3125.5	2302.8	3.18
Halosphaeriaceae	0.0096	108.29	2500.9	2217	103.37	900.26	2569.4	3.09
Bionectriaceae	0.0049	0	1418.9	2385.4	1580.6	205.95	444.84	3.08
Acaulosporaceae	0.0006	2114.1	0	666.4	0	0	0	3.02
Trichosporonaceae	0.0238	0	706.71	851.14	0	0	2115	3.02
Sordariaceae	0.0325	920.85	1056.8	2013.6	1279.2	0	751.86	3
Olpidiaceae	0.0033	1963.2	1326.2	0	705.27	483.51	0	2.99
Agaricaceae	0.0024	0	201.54	1046.2	0	1584.2	1822.1	2.96
Plectosphaerellaceae	0.0035	0	201.54	663.13	95.411	0	1737.1	2.94
Pseudoplagiostomataceae	0.0016	0	0	0	1715.7	295.87	313.9	2.93
Dipodascaceae	0.0005	0	218.47	0	0	1175.4	1613.6	2.91
Bolbitiaceae	0.0011	0	0	670.8	95.411	0	1598.4	2.9
Dothioraceae	0.0005	0	1438.4	0	0	0	1186.4	2.86
Apiosporaceae	0.0008	1281.8	0	94.733	0	789.73	108.21	2.81
Gnomoniaceae	0.0032	0	0	0	1103.6	303.38	309.2	2.74
Total abundance		1406722	6298124	4265498	870721	1957812	3940315	
Selected bacteria/total bacteria		14.07%	62.98%	42.66%	8.71%	19.58%	39.40%	

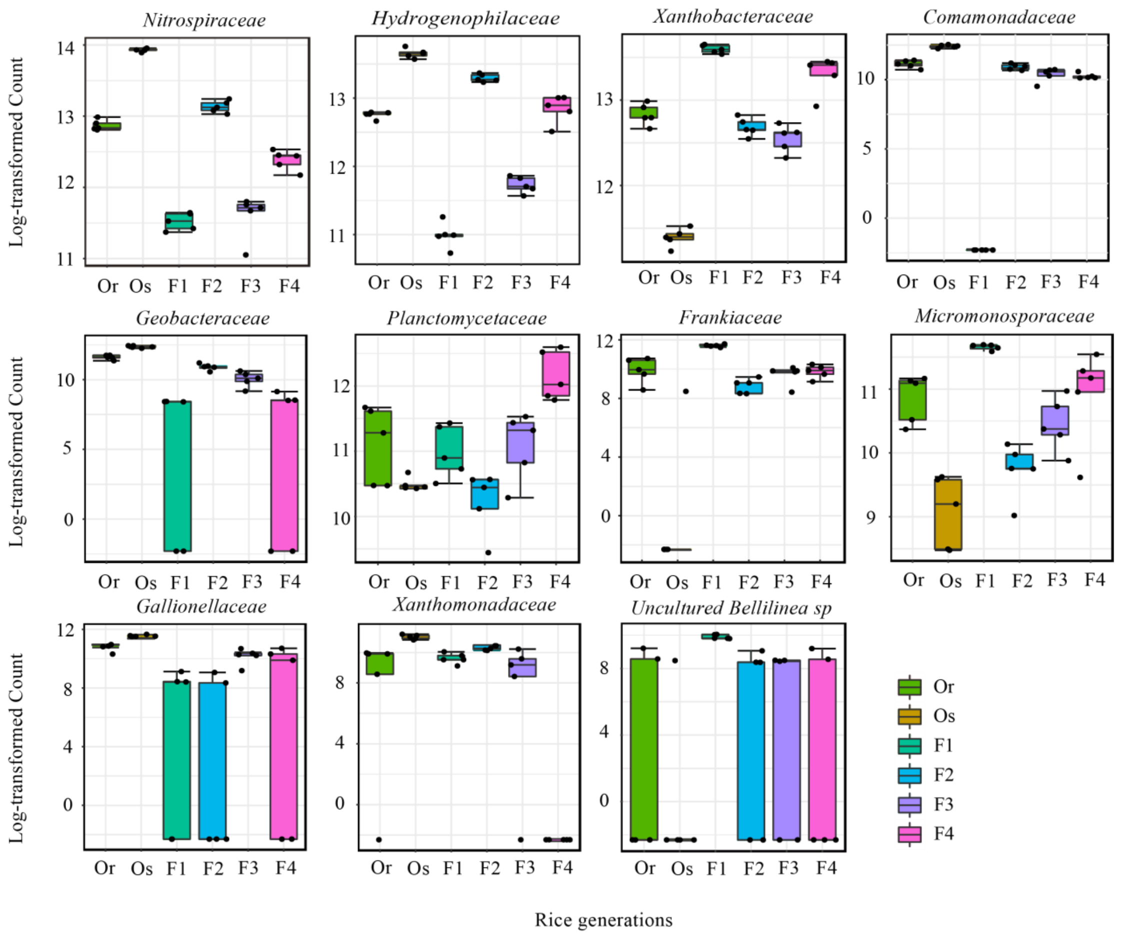

Figure S1. Rhizosphere bacterial communities (family level) affected by wild rice *Oryza rufipogon* (Or) female parent. Rhizosphere of parental wild rice *Oryza rufipogon* (Or), parental cultivated rice *Oryza sativa* (Os) and different hybrid generations of rice progenies (F1) obtained by self-crossing (F2, F3, F4).

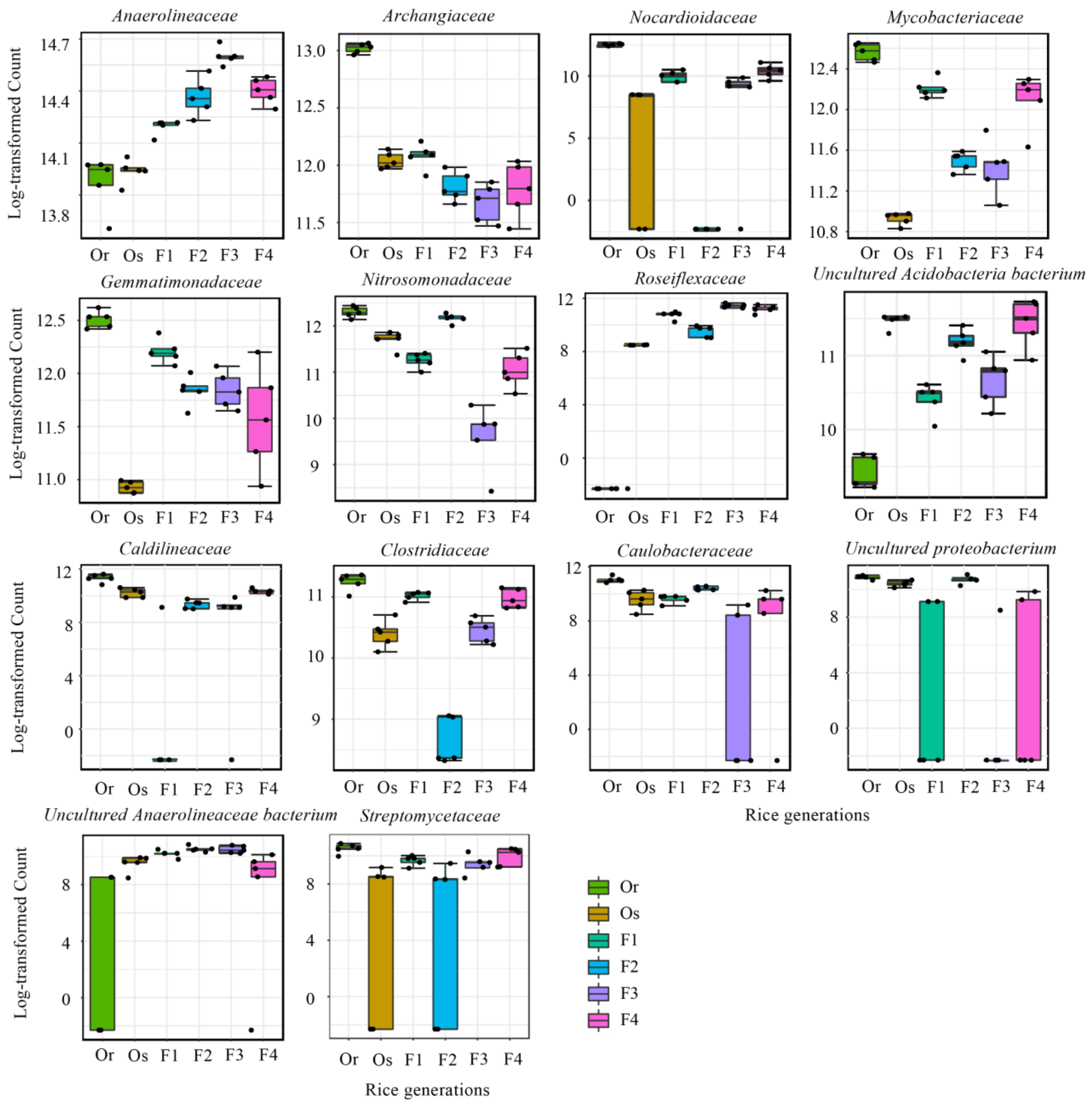

Figure S2. Rhizosphere bacterial communities (family level) affected by cultivated rice *Orysa sativa* (Os) male parent. Rhizosphere of parental wild rice *Oryza rufipogon* (Or), parental cultivated rice *Oryza sativa* (Os) and different hybrid generations of rice progenies (F1) obtained by self-crossing (F2, F3, F4).

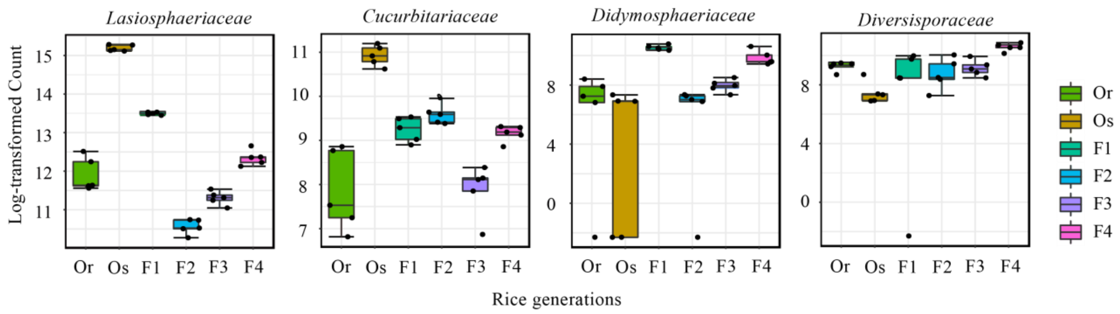

Figure S3. Rhizosphere fungal communities (family level) affected by parental wild rice *Oryza rufipogon* (Or). Rhizosphere of parental wild rice *Oryza rufipogon* (Or), parental cultivated rice *Oryza sativa* (Os) and different hybrid generations of rice progenies (F1) obtained by self-crossing (F2, F3, F4).

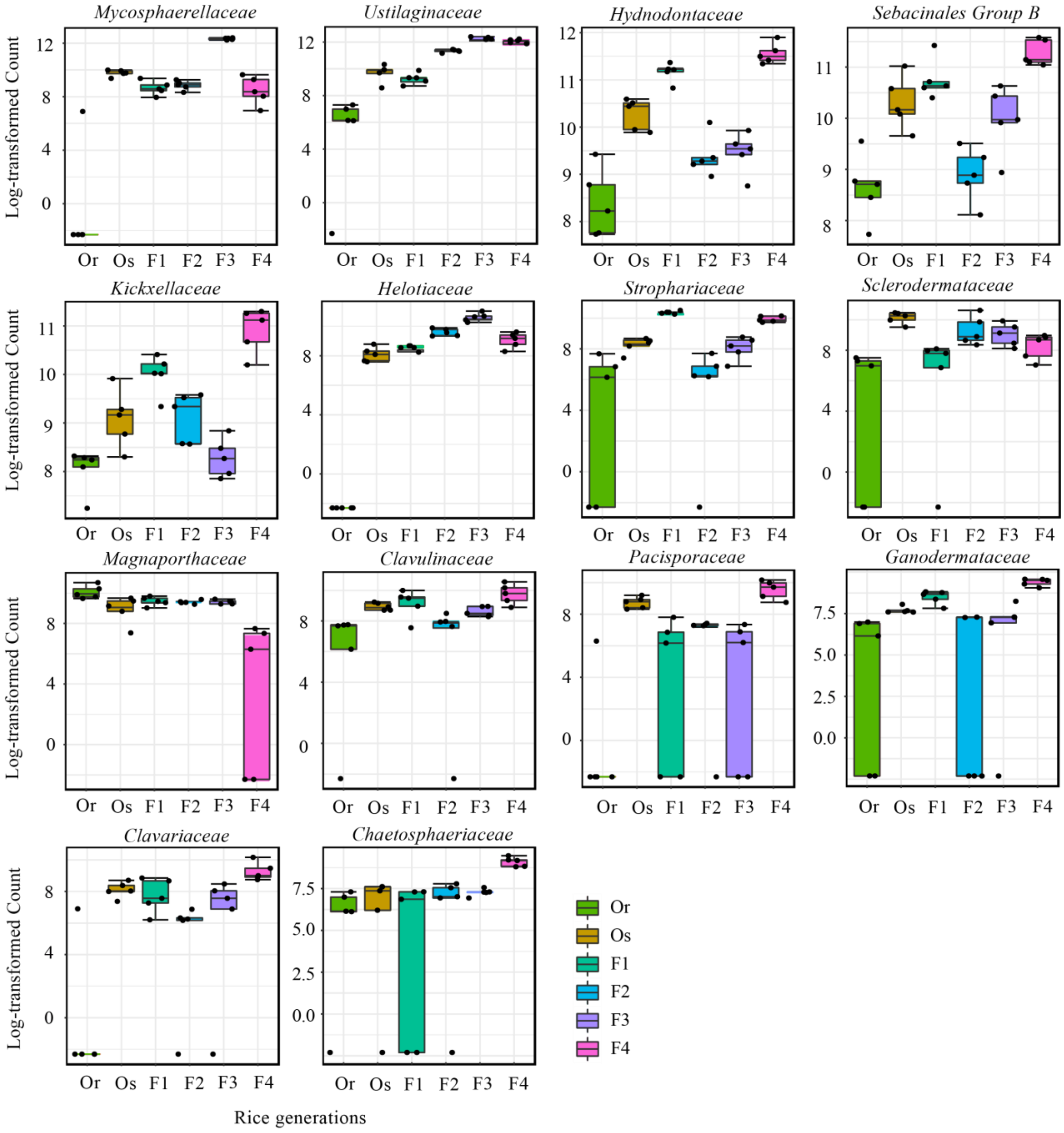

Figure S4. Rhizosphere fungal communities (family level) affected by parental cultivated rice *Oryza sativa* (Os). Rhizosphere of parental wild rice *Oryza rufipogon* (Or), parental cultivated rice *Oryza sativa* (Os) and different hybrid generations of rice progenies (F1) obtained by self-crossing (F2, F3, F4).

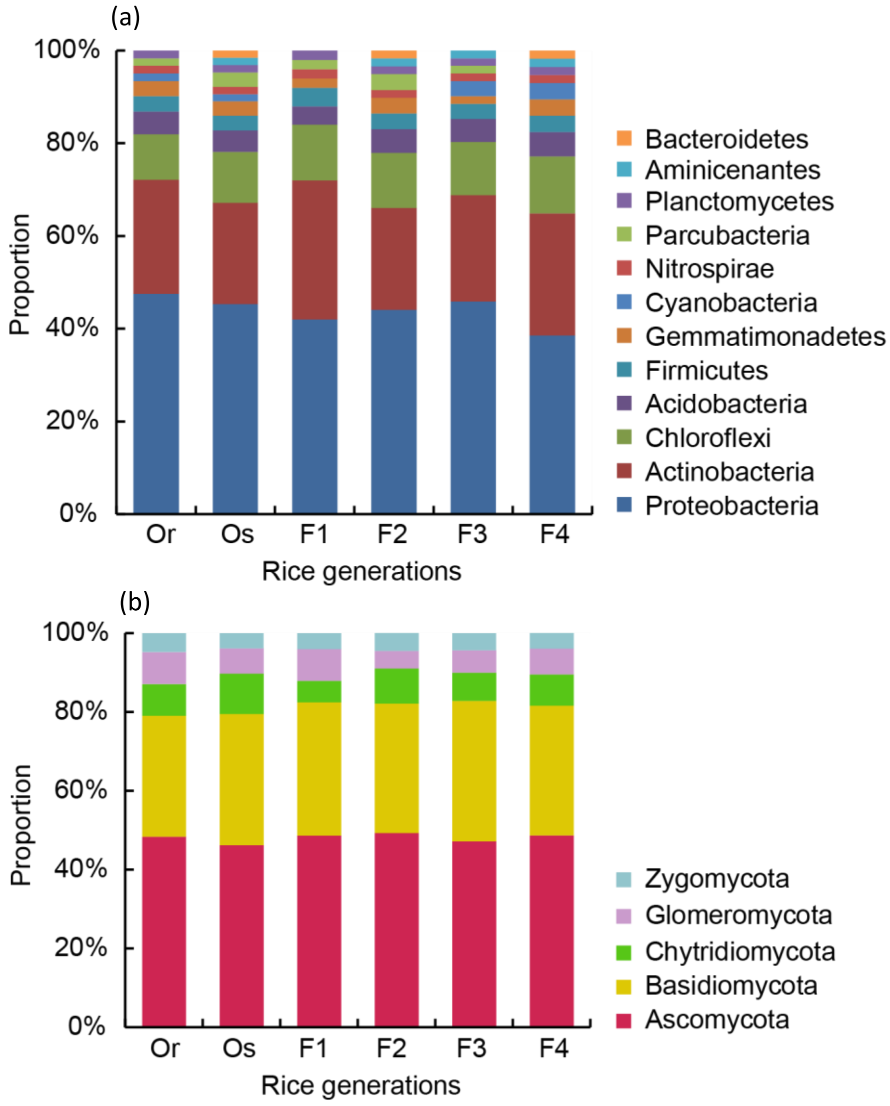

Figure S5. The proportion of the key bacteria family (a) and fungi family (b) affiliated to phylum. Rhizosphere of parental wild rice *Oryza rufipogon* (Or), parental cultivated rice *Oryza sativa* (Os) and different hybrid generations of rice progenies (F1) obtained by self-crossing (F2, F3, F4).
